# Issue on general and abdominal surgery – an exciting and challenging surgical discipline in the spectrum of operative medicine

**DOI:** 10.1515/iss-2023-0053

**Published:** 2023-11-13

**Authors:** Karsten Ridwelski, Frank Meyer

**Affiliations:** Institute of Quality Assurance in Operative Medicine (IQOM), Otto von Guericke University, Magdeburg, Germany; Department of General and Abdominal Surgery, Municipal Hospital (Klinikum Magdeburg GmbH), Magdeburg, Germany; Department of General, Abdominal, Vascular and Transplant Surgery, Otto von Guericke University with University Hospital, Magdeburg, Germany

Dear Readers,

Welcome to the current issue of *Innovative Surgical Sciences* on the subject area General and Abdominal Surgery. The Guest Editors believe that they were able to generate an exciting issue. Let us invite you to take your time, to linger on its content and get your own impression.

As you – the reader – may assess and monitor, it is an overwhelming challenge to create an interesting issue of the journal with the given subtitle including the hot topics of the specific surgical subspecialty named General Surgery, which also includes – according to the understanding of the Guest Editors – aspects of abdominal surgery. 

One of the articles, namely “Hajduk E *et al*. on “Does intestinal anastomosis in resection of colon cancer have a significant impact onto early postoperative outcome and long-term survival?” on quality assurance in operative medicine - a main range of topics of this issue - is dedicated to Prof. Dr. Hans Lippert (Magdeburg), one of the German protagonists out of this specific field of clinical research on clinical care (see the references list).

Therefore, a representative mixture of

(i) variable article types such as (Narrative) Review (Andric et al., Brinkers et al., Kraus et al.; Meißner et al.), Original Article (Arndt M et al., Hajduk et al.; Meißner et al.) and Case Reports (Perrakis et al., Arndt S et al.*, Lorenz et al.),
(ii) methodological aspects such as Clinical Prospective Observational Studies (Arndt M et al., Hajduk et al., Meißner et al.), rare cases/case constellations (Arndt S et al.*, Lorenz et al., Perrakis et al.) as well as
(iii) diverse subtopics, such as rare indication for liver transplantation (Lorenz et al.), conservative therapeutic options in acute appendicitis (Andric et al.), “oncosurgery” (Arndt M et al., Hajduk et al., Perrakis et al.), colorectal surgery (Arndt M et al., Hajduk et al., Perrakis et al.) and prehabilitation (Meißner et al.) was chosen and favored.

The various dispositions reflect vividly the broad spectrum of the topics. The topics were mainly suggested by the Guest Editors and were obtained from active clinical researchers and surgical colleagues of the field.

As selected commentary:

Studies on quality assurance in clinical medicine, in particular, in “oncosurgery”, have a long tradition at the Magdeburg’s Institute of Quality Assurance in Operative Medicine (Otto von Guericke University; former scientific heads: Profs. Lippert and Gastinger) - see a selection in the reference list [1], [2], [3], [4], [5], [6], [7].

This issue presents the two latest studies, namely on multivisceral resection (MVR) in colon and rectum cancer (CA), which allows curation by R0 resection with adequate long-term survival. In detail (as an excerpt from the article to arouse the reader’s interest), “MVR tended to be associated with reduced 5-year overall survival rates (significant only for pT4 colon CA based on the matched-pair analysis [MPA] results), as well as, with a significant increase in morbidity rates in both tumor entities. In the overall data, MVR was associated with significant increases in hospital lethality rates, as indicated by the MPA (significant only for rectal CA)”.

In addition, the variable impact of nutritional aspects (Meißner et al.) and of anastomosis in colorectal tumor surgery (Hajduk et al.) is demonstrated – see below.

In an issue on daily topics of general and abdominal surgery, a (narrative) review on current aspects in an appropriate stage-, finding- and inflammatory severity-adapted management of acute appendicitis may not be lacking. It seems that there are certain characteristics which may allow to decide for a conservative approach (with reliable issues provided by transabdominal ultrasound for diagnostics) or, in contrast, to set a strict indication for surgery (e.g., in case of [a] detected appendicolith[s]).

A great ambition of the Guest Editors is the well-developed collaboration of general and abdominal surgery with other clinical, conservative, surgical or operative disciplines, represented in this issue by topics, such as on “What must the abdominal surgeon need to know on plastic surgery” (Kraus et al.) and pain therapy (Brinkers et al.).

The illustrative collection of topics and various manuscript types is completed by three case reports (again, Arndt S et al.*, Perrakis et al., Lorenz et al.), a usually underestimated manuscript category but with a high educational level:

– first, on necessary laparoscopic cholecystectomy for symptomatic cholecystolithiasis (CCL) in case of a Kaposi-like tumor lesion, the Kasabach-Merritt syndrome (KMS), which is characterized by pronounced veins at various regions of the body, which can make the surgical approach very challenging;
– second, on the rare colorectal malignant tumor site *ex ano*, with an impressive manifestation among the most frequent malignant tumor lesions;
– third, on a rare indication for liver transplantation due to liver failure caused by heat shock.

Hopefully you will like the meaningful introduction provided by this Editorial and you have already become curious on the current issue’s content. Yes, please, let us know your impressions using reader’s “Letters to the Editor“ or “Short Communication“ as appropriate.

We would be glad if you have a great time in reading the favored articles chosen and presented.

Many thanks to the Editor-in-Chief, Prof. Jähne, for the magnificent opportunity in arranging this colorful collection of articles as well as for favoring our attitude, ideas and guest editing as well as selections of authors, topics and written papers. Many thanks also to the Managing Editor, Dr. S. Sporn, for support in the manuscript processing, galley proofs and final version of the journal‘s issue.

Last but not least, we are grateful for the great input of all authors.

Karsten Ridwelski and Frank Meyer

Guest Editors.
